# Impaired lipid metabolism in idiopathic pulmonary alveolar proteinosis

**DOI:** 10.1186/1476-511X-10-54

**Published:** 2011-04-12

**Authors:** Xinlun Tian, Jinmei Luo, Kai-Feng Xu, Lan Wang, Jiong Zhou, Ruie Feng, Yaosong Gui, Juan Wang, Wenbing Xu, Yi Xiao, Yuanjue Zhu

**Affiliations:** 1 Department of Respiratory Medicine, Peking Union Medical College Hospital, Chinese Academy of Medical Sciences & Peking Union Medical College, Beijing, 100730, China; 2 Office for Infection Control, Peking Union Medical College Hospital, Chinese Academy of Medical Sciences & Peking Union Medical College, Beijing, 100730, China; 3 Department of Pathology, Peking Union Medical College Hospital, Chinese Academy of Medical Sciences & Peking Union Medical College, Beijing, 100730, China

**Keywords:** high-density lipoprotein cholesterol, lipid, pulmonary alveolar proteinosis, triglyceride

## Abstract

**Background:**

It is well known that lipids abnormally accumulate in the alveoli during idiopathic pulmonary alveolar proteinosis (PAP). It is unclear, however, whether lipids also abnormally accumulate in serum. This study investigated the serum lipid panels in idiopathic PAP patients and explored the relationships between serum levels and the severity of idiopathic PAP.

**Methods and Results:**

Clinical data including the level of serum lipids were evaluated in 33 non-diabetic idiopathic PAP patients and 157 healthy volunteers. Serum levels of triglyceride were higher in PAP patients than in healthy subjects (median: 192.00 mg/dl (*P*_25_: 104.36, *P*_75_: 219.00) *vs *119.56 mg/dl (*P*_25_: 78.81, *P*_75_: 193.03), *P *< 0.05), while high-density lipoprotein cholesterol (HDL-C) levels were lower in patients than in the control group (42.50 ± 10.30 *vs *51.34 ± 12.06 mg/dl, *P *< 0.01). Forced expiratory volume in one second and forced vital capacity in hypertriglyceridemia patients were lower than those in patients with normal triglyceride. Serum LDL-C and HDL-C ratio correlated negatively with PaO_2 _(r = -0.403, *P *< 0.05) and positively with lactate dehydrogenase (r = 0.381, *P *< 0.05).

**Conclusions:**

PAP associates with high triglyceride and low HDL levels in the serum, and these lipids provide potential intervention strategy for treatment.

## Introduction

Pulmonary alveolar proteinosis (PAP) is a rare lung disease in which the alveolar spaces are filled with lipoproteinaceous material [1,2]. PAP occurs in three clinically distinct forms: congenital, secondary, and idiopathic [1,3,4]. More than 90% of PAP patients are idiopathic [2]. Most patients with PAP are symptomatic with progressive exertional dyspnea of insidious onset and cough, and are hypoxemic in room air. A primary complication of PAP is that it predisposes patients to pulmonary infections. Disease outcomes vary from the development of respiratory failure to full symptom resolution. The primary treatment strategy for idiopathic PAP is whole lung lavage.

Bronchoalveolar lavage fluid from PAP patients shows a milky appearance compared with control samples and contains large, foamy alveolar macrophages along with an increased number of lymphocytes. The opacity is due to an increased surfactant concentration, which has been shown to accumulate due to reduced clearance kinetics rather than increased synthesis rates. Surfactant, a complex mixture of phospholipids and proteins, serves to reduce surface tension within the lung. Cholesterol is the most abundant neutral lipid of pulmonary surfactant, constituting up to 90% of the neutral lipid materials [1,5]. At least 80% of the cholesterol present in the lung, and virtually all that in surfactant, is derived from circulating lipoproteins, with very low-density lipoprotein believed to be the major vehicle of delivery to the lung [6]. Because patients with PAP have increased lipoproteins in the lung, we hypothesized that circulating lipoprotein levels would also be changed.

There is little information on the association between serum lipids, lipoprotein levels, and PAP severity. The current study investigated these relationships to determine whether serum lipid levels would provide valuable clinical information to assess and monitor disease progression.

## Methods

### Study design and population

The institutional review board committee of Peking Union Medical College Hospital approved the protocol of this study. Data from healthy volunteers were obtained from persons who were examined in the Center of Medical Examination in Peking Union Medical College Hospital between April 2009 and May 2010. All study participants provided informed consent. Data for the PAP patients were obtained retrospectively from the case records. Using a case-controlled study design, data of 157 previously healthy persons were evaluated for the level of lipids in a non-diabetic population and compared to the idiopathic PAP cohort. There were 80 PAP patients diagnosed in our hospital between January 2001 and February 2009. Complete datasets, including lipid analysis and other criteria, were obtained from 33 patients at the time of diagnosis and were used in this analysis. We included only idiopathic PAP patients with normal fasting serum glucose levels (< 7.0 mmol/L) to avoid potential confounding effects of diabetes mellitus on serum lipid metabolism. The diagnosis of idiopathic PAP was established by histopathologic examination of material from open lung biopsies, transbronchial biopsies or bronchial alveolar lavage, and by exclusion of secondary etiologies by history and laboratory investigation. Anti-GM-CSF antibodies were not measured in this study because blood samples were not available during the retrospective analysis. Patients were excluded if they had a history of diabetes mellitus, chronic liver or kidney disease, infection, cancer, or use of corticosteroids or lipid-lowing agents. Detailed demographic, medical history, history of smoking, body mass index (BMI), blood pressure, fasting serum glucose, lipid levels (total cholesterol (TC), triglyceride (TG), low-density lipoprotein cholesterol (LDL-C), high-density lipoprotein cholesterol (HDL-C)), pulmonary function tests (including forced expiratory volume in one second (FEV_1_), forced vital capacity (FVC), FEV_1_/FVC, total lung capacity (TLC) and carbon monoxide diffusion capacity (DLco), arterial blood gas analysis in room air, and treatment were all recorded and entered into a database.

Hypertriglyceridemia (hyper-TG) was defined as serum TG ≥150 mg/dl. Hyper-low-density-lipoproteinemia (hyper-LDL) was defined as serum LDL-C ≥130 mg/dl. Hypercholesterolemia (hyper-TC) was defined as serum TC ≥200 mg/dl. Hypo-high-density-lipoproteinemia (hypo-HDL) was defined as serum HDL-C ≤40 mg/dl based on the China's guidelines of prevention and treatment of adult dyslipidemia [7].

### Assays

Laboratory examinations were performed on blood samples obtained after an overnight fasting. The levels of serum TC, TG, HDL-C and LDL-C, fasting serum glucose, and lactate dehydrogenase (LDH) were measured with commercial kits using an automated chemistry analyzer (Olympus AU5400, Japan). All arterial blood samples were collected with the patient breathing room air and were used to calculate alveolar-arterial oxygen pressure difference (P(A-a)O_2_) (Radiometer ABL835, Denmark). Pulmonary function tests were performed with the Master Screen Body equipment (Jaeger, Germany) using current recommendations of the ATS/ERS Task Force on standardizations of pulmonary function tests [8-10]. The values were expressed as a percentage of the predicted values for gender, age and height. Data of DLco was normalized to hemoglobin levels.

### Statistical analysis

Statistics were performed with SPSS version 11.0. Normality of variables was tested by the Kolmogorov-Smirnov test. An unpaired, two tailed t-test and a Chi-squared analysis was used for comparison between the two groups. The nonparametric Mann-Whitney U-test was used when data were not normally distributed. Specifically, TG data in this group and also in the general population was not normally distributed and was evaluated by the nonparametric test. A *p*-value of <0.05 was considered statistically significant.

## Results

In the PAP group, there were 23 men and 10 women with a mean age of 43.9 years old. Seven subjects had a history of cardiovascular disease, cerebral vascular disease, or hypertension, while fifteen subjects had a history of smoking, and six patients had previously accepted sole or whole lung lavage therapy.

The patient characteristics of the PAP group were compared with those of the healthy control group, as shown in Table 1. There were no significant differences in age, BMI, and gender between the two groups (*p *= not significant (NS)). Furthermore, no significant difference was found in the smoking status, cardiovascular disease, cerebral vascular disease, or hypertension (p = NS). The mean systolic blood pressure (SBP), diastolic blood pressure (DBP), and fasting serum glucose levels were all within normal ranges, and there was no significant difference between the two groups (p = NS).

**Table 1:** Characteristics of the patients and healthy subjects

	Control (n = 157)	Patients (n = 33)	***P***
Sex (male/female)	84/73	23/10	0.088
Mean age (yrs)	41 ± 12	44 ± 9	0.195
BMI (kg/m^2^)	24.6 ± 3.6	23.7 ± 3.7	0.223
Cardiovascular history	19/144 (13.2%)	7/33 (21.2%)	0.368
Smoking status:yes/total (%)	48/144 (33.3%)	15/33 (45.5%)	0.19
Systolic blood pressure (mmHg)	122 ± 14	121 ± 18	0.817
Diastolic blood pressure (mmHg)	78 ± 10	79 ± 13	0.727
Fasting serum glucose (mmol/l)	4.67 ± 0.44	4.66 ± 0.64	0.936
TC (mg/dl)	191.24 ± 35.60	198.87 ± 43.16	0.283
TG (mg/dl)*	118.65(78.81-193.03)	192.00(104.36-219.00)	0.011
HDL-C (mg/dl)	51.34 ± 12.06	42.56 ± 10.30	<0.001
LDL-C (mg/dl)	119.56 ± 32.04	133.41 ± 35.54	0.03
LDL/HDL	2.46 ± 0.87	3.30 ± 1.17	<0.001
TG/HDL	3.28 ± 2.88	4.75 ± 3.11	0.001
TC/HDL	3.90 ± 1.05	4.89 ± 1.41	<0.001
Hb (g/L)	145.28 ± 15.01	158.85 ± 22.66	<0.001
FEV_1 _pred (%)	93.74 ± 13.33	76.79 ± 19.28	<0.001
FVC pred (%)	96.80 ± 12.37	75.98 ± 19.97	<0.001
FEV_1_/FVC (%)	83.12 ± 6.74	83.54 ± 6.53	0.757

Values are expressed as the mean ± SD unless otherwise indicated. *: Values are expressed as median, 25% and 75%. Nonparametric Mann-Whitney U-test was used, as data were not normally distributed.

Serum levels of TG (median: 192.00 mg/dl (*P*_25_: 104.36, *P*_75_: 219.00) *vs *118.65 mg/dl (*P*_25_: 78.81, *P*_75_: 193.03), *p *= 0.011) and LDL-C (133.41 ± 35.54 *vs *119.56 ± 32.04 mg/dl respectively, *p *= 0.03) were higher in PAP patients than healthy subjects, while HDL-C levels were lower in patients than those in controls (42.56 ± 10.30 mg/dl *vs *51.34 ± 12.06 mg/dl, *p *< 0.001). In patients and healthy subjects, the TC/HDL-C ratios were 4.89 ± 1.41 and 3.90 ± 1.05, respectively (*p *< 0.001), LDL-C/HDL-C ratios were 3.30 ± 1.17 and 2.46 ± 0.87 (*p *< 0.001), and TG/HDL-C ratios were 4.75 ± 3.11 and 3.28 ± 2.88 (*p *= 0.001). However, the levels of TC were similar in the two groups (p = NS).

Lipid metabolism is influenced by many factors, thus, we compared the effects of PAP, gender, age, BMI, patient history, smoking, and blood glucose on triglyceride and HDL-C levels by logistic regression. Only PAP and BMI were significantly associated with triglyceride and HDL-C levels. PAP did not have effects on LDL-C and TC levels in this analysis.

According to the definitions of hyper-TG, hyper-LDL, hyper-TC, and hypo-HDL, patients were divided into hyper-, hypo-, or normal groups. We found that PAP patients were more likely to be in the hyper-TG group compared to the healthy control group (OR = 2.32, 95% CI 1.08-4.97, *p *= 0.028). PAP patients were also more likely to be in the hypo-HDL group compared to those in the control group (OR = 5.40, 95% CI 2.37-12.32, *p*ä0.001). The hyper-TC and hyper-LDL comparisons did not show any differences between PAP patients and the control group (OR = 1.46, 95% CI 0.69-3.12, *p = NS*; OR = 2.04, 95% CI 0.95-4.40, *p *= NS, respectively).

To identify relationships between abnormal lipid levels and PAP severity, we compared the partial pressure of arterial oxygen (PaO_2_), LDH, and pulmonary function tests of the hyper-TG PAP group and the normal TG PAP group. We found that in the hyper-TG PAP group, FEV_1 _and FVC were lower than those of the normal TG PAP group (p < 0.05 for both). TLC and DLco were not significantly different between the two groups (Table 2).

**Table 2:** Comparison of clinical features between hyper-TG and normal TG PAP patients

	Normal TG patients (N = 14)	Hyper-TG patients (N = 19)	***P***
Sex (male/female)	11/3	12/7	0.455
Age (yrs)	43.79 ± 8.26	43.89 ± 8.91	0.971
BMI (kg/m^2^)	24.4 ± 5.00	23.20 ± 2.55	0.417
Hypertension history (yes/no)	2/12	5/14	0.67
Smoking history (yes/no)	8/6	7/12	0.304
Systolic blood pressure (mmHg)	119 ± 16	123 ± 19	0.547
Diastolic blood pressure (mmHg)	77 ± 13	80 ± 14	0.606
Fasting serum glucose (mmol/l)	4.83 ± 0.77	4.54 ± 0.50	0.21
Hb (g/L)	152.07 ± 22.11	163.84 ± 22.30	0.143
LDH (U/l)	295.85 ± 118.42	257.16 ± 91.10	0.305
PaO_2_	65.42 ± 22.08	66.18 ± 12.34	0.912
P_A-a_O_2 _(mmHg)	40.00 ± 21.55	39.20 ± 12.67	0.897
FEV1 pred (%)	87.73 ± 15.70	70.71 ± 18.71	0.022
FVC pred (%)	87.32 ± 16.44	69.67 ± 19.31	0.022
FEV1%	82.70 ± 5.8	84.01 ± 7.00	0.620
TLC pred (%)	79.89 ± 10.99	75.43 ± 12.83	0.373
DLco pred (%)	67.80 ± 13.80	60.15 ± 16.17	0.255

Values are expressed as the mean ± SD.

**Figure 1:**
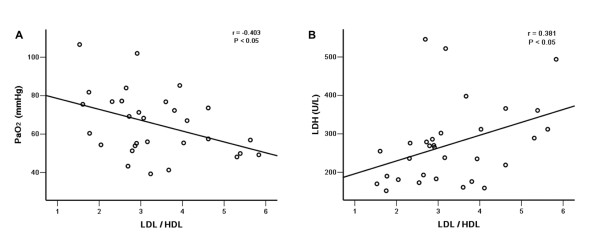
**Correlations between serum LDL/HDL ratio and PaO_2 _(A) or LDH (B)**. Serum LDL-C/HDL-C ratios correlated negatively with PaO_2 _levels (r = -0.403, *p *= 0.027) and positively with LDH (r = 0.381, *p *= 0.034), n = 33.

Correlations between serum LDL-C/HDL-C ratios and PaO_2 _or LDH levels are shown in Figure 1. Serum LDL-C/HDL-C ratios correlated negatively with PaO_2 _levels (r = -0.403, *p *= 0.027) and positively with LDH (r = 0.381, *p *= 0.034). Serum LDL-C/HDL-C ratios did not correlate with P(A-a)O_2 _levels (r = 0.335, *p *= 0.070). Moreover, there was no linear correlation between serum lipids and FEV1 or FVC.

## Discussion

Multiple studies have focused on lipid metabolism disorders in coronary vascular disease, diabetes mellitus, obesity, and atherosclerosis. A growing body of literature suggests that metabolic syndromes could also be present in patients with a variety of pulmonary diseases, including pulmonary arterial hypertension [11] and lung fibrosis [12]. PAP, also referred to as alveolar lipoproteinosis, is an enigmatic disorder characterized by abnormal intra-alveolar surfactant accumulation. Elevated serum cholesterol levels have been reported in 19% of PAP patients, however, sample size and characteristics were not described [1]. Absolute levels of cholestenoic acid were significantly increased in the serum of PAP patients compared with those of controls [13]. Moreover, Inoue *et al. *showed that hyperlipemia occurs in 4.2% of Japanese idiopathic PAP patients [14], but detailed lipid profiles were not described. This led us to hypothesize that serum lipoproteins could also be elevated in PAP patients.

Our data shows that serum lipid level differences did associate with PAP, as TG levels are higher and HDL-C levels are lower in PAP patients compared to those in the normal control group. PAP patients with hyper-TG had lower FEV1 and FVC than PAP patients with normal TG levels. In our study, the mean BMI in the PAP group was below 25 kg·m^-2^, which is within the normal weight range. We also excluded patients with diabetes or patients using corticosteroids or lipid-regulating drugs. Blood pressure is also matched between the two groups. Therefore, we can conclude that the hyperlipemia associated with PAP is likely an independent factor. Of interest, total triglyceride, but not total cholesterol, in our PAP group is elevated.

Although the etiology of PAP remains unclear, recent studies into PAP pathogenesis have revealed the important roles for GM-CSF [2,5,15]. Alveolar macrophages from GM-CSF gene knockout mice have reduced capacity for surfactant catabolism [2,16]. Studies have shown that exogenous administration of GM-CSF benefits some PAP patients, which supports the potential of GM-CSF as a replacement therapy for PAP [17-19].

The mechanisms of dyslipoproteinemia in PAP are unknown, but may occur through GM-CSF roles. GM-CSF can lower plasma cholesterol and plasma triglycerides levels, elevate expression of very-low density lipoprotein receptors, decrease scavenger receptor expression on human macrophages, and attenuate the accumulation of cholesterol esters [20]. GM-CSF is also a pro-inflammatory cytokine, and recent evidence suggests that chronic inflammatory diseases associate with metabolic syndrome and insulin resistance [21,22]. GM-CSF increases tumor necrosis factor-α and interleukin-1β gene expression, which could affect lipid metabolic disorders [21,23,24].

In addition, peroxisome proliferators-activated receptor-γ (PPAR-γ) mRNA and protein are highly expressed in alveolar macrophages in normal subjects [25,26]. In PAP patients, both PPAR-γ mRNA and the PPAR-γ-regulated lipid scavenger receptor CD36 in alveolar macrophages are reduced. In PAP patients, GM-CSF treatment increased PPAR-γ to control levels [25,27]. In addition to serving as a potential factor in GM-CSF regulation of lung homeostasis, PPAR-γ is also an important transcriptional regulator of genes involved in glucose and lipid metabolism [28]. Restoration of PPAR-γ reduced lipid accumulation in alveolar macrophages of GM-CSF knockout mice [26]. PPAR-γ agonists are commonly used in the treatment of diabetes, and have effects in lowering plasma TG and LDL-C levels, and increasing HDL-C levels [29,30]. Hence, PPAR-γ may partially be involved in lipid metabolism in PAP.

We found that hyper-TG correlated with lower FEV_1 _and FVC. Serum LDL-C/HDL-C ratios correlated negatively with PaO_2 _levels and positively with LDH. Previous studies have demonstrated a correlation between LDH and PaO_2 _[1,31]. In our study, TG levels did not correlate with LDH levels, which is a major biomarker currently available for PAP severity. Therefore, whether the severity of PAP is related to the degree of dyslipoproteinemia remains to be clarified.

From this study, we recommend that serum lipid analysis be included in the evaluation of PAP patients. The possibility that PAP patients may be at a higher risk for coronary heart disease should also be evaluated. Furthermore, our study suggests that lipid-modifying drugs may be a potential novel intervention for PAP patients.

While the present findings are interesting, our study has several limitations. The majority of our patients were not re-evaluated for lipid levels when they were followed up at the outpatient clinics. Therefore, we could not ascertain whether lipid levels are higher in patients during active disease compared to remission. A prospective and comprehensive study to validate the role of circulating lipids would be required. A prospective study design would also allow anti-GM-CSF antibodies to be measured, to clarify the idiopathic auto-immune PAP from secondary PAP and to determine the relationship of titration of anti-GM-CSF and lipid abnormalities. Hypertriglyceridemia and hypo-high-density-lipoproteinemia are more prevalent in non-diabetic idiopathic PAP patients than in healthy controls. While the etiology of PAP is likely to be multifactorial, further studies are warranted to determine the potential benefits of lipid monitoring and intervention for PAP patients.

## Competing interests

The authors declare that they have no competing interests.

## Authors' contributions

XT, KFX and YZ designed the study. LW, JZ and YG carried out the statistical analysis. RF reviewed the pathological results. JW carried out pulmonary function tests. All authors carried out data collection. XT, JL and KFX wrote the manuscript. All authors read and approved the final manuscript.
